# Psychometric evaluation of a structured assessment tool for nurse anesthetists’ non-technical skills

**DOI:** 10.1186/s12909-025-07297-2

**Published:** 2025-05-17

**Authors:** Martin Jarl, Cecilia Escher, Piotr Harbut, Helen Conte, Ulrica Nilsson

**Affiliations:** 1 Department of Neurobiology, Care Sciences and Society, Karolinska Institute, Alfred Nobels Allé 23, Stockholm, 171 77 Sweden; 2 Department of Anesthesia and Intensive Care, Danderyd University Hospital, Stockholm, Sweden; 3 Department of Anesthesia and Intensive Care, Norrtälje Hospital, Norrtälje, Sweden; 4 Department of Clinical Science, Intervention and Technology-CLINTEC, Karolinska Institutet, Stockholm, Sweden; 5 Department of Clinical Sciences, Danderyds Hospital, Karolinska Institutet, Stockholm, Sweden

**Keywords:** Non-technical skills, Nurse Anesthetist, NANTS, Psychometric, Training, Assessment, Behavior Marker System, Professional Development, Clinical Competency

## Abstract

**Background:**

Non-technical skills are the essential cognitive, social, and personal resources contributing to safe and efficient task performance. An assessment tool can facilitate the development and teaching of non-technical skills. The nurse anesthetist non-technical skills tool includes four categories and fifteen elements and is an adaptation of the existing tools for physician anesthetists and Danish nurse anesthetists. The ratings are on a five-step scale, with an option to select “Not Relevant”. Since there doesn’t exist an assessment tool for Swedish nurse anesthetists’ non-technical skills, the aim of the study was to translate and adapt the assessment tool for nurse anesthetists’ non-technical skills to a Swedish context and test its psychometric qualities among nurse anesthetists with experience in teaching nurse anesthetist students and junior nurse anesthetists in clinical settings.

**Methods:**

In this prospective psychometric evaluation study, sixteen nurse anesthetists were recruited. They rated 12 video clips of simulated anesthesia scenarios after participating in a three-hour calibration workshop. Four weeks later, a test–retest was conducted, which included five video clips. Internal consistency, Interrater reliability, and test–retest reliability were examined.

**Results:**

Internal consistency showed acceptable results on the element level and Interrater reliability indicated good results. Retest reliability showed poor to moderate reliability. The use of “Not Relevant” varied significantly depending on the length of the video clip and the provider being rated. The raters considered the assessment tool suitable but initially challenging to use for rating non-technical skills among nurse anesthetists and articulate non-technical skills in anesthesia nursing.

**Conclusions:**

This initial testing of the Swedish nurse anesthetists’ non-technical skills tool shows acceptable psychometric qualities and gives a foundation for future research. However, the rating “Not Relevant” poses challenges that need to be addressed. Nevertheless, the participants consider the assessment of non-technical skills in Swedish nurse anesthetists to be appropriate.

**Supplementary Information:**

The online version contains supplementary material available at 10.1186/s12909-025-07297-2.

## Background

Non-technical skills (NTS) are recognized by experts [1] and government authorities [2, 3] as an important contributor to safe patient care. NTS are a part of human factors [1] and enhance technical skills and clinical knowledge by minimizing the effect of human behavior on adverse events [4]. Non-technical skills are defined as *“the cognitive, social and personal resource skills that complement technical skills, and contribute to safe and efficient task performance” *[4].

Anesthetists are the primary anesthesia providers in most countries, but in some countries, nurse anesthetists (NA) or anesthesia assistants are used with varying degrees of supervision [5]. In the Nordic countries, NA is qualified to conduct anesthesia for healthy patients, independently with indirect supervision from an anesthetist [6, 7]. Surgery and anesthesia take place in a complex environment, and a routine operation can quickly develop into a surgical emergency requiring time-sensitive decision-making, and effective communication within the operation room (OR) team. This requires the NA to be vigilant and carefully monitor the patient's condition in order to maintain situational awareness [1]. McCulloch et al. [8] trained OR teams in NTS and observed a decrease in both surgical technical errors, procedural errors outside the surgical field and an increase in the teams’ attitudes to patient safety. During medical emergencies, quick and correct decisions are vital for solving the medical crisis, which is facilitated by high levels of NTS [1]. Teams with high levels of NTS have been linked to resolving surgical and anesthesia crises faster during simulations [9] as well in obstetric teams during major post-partum hemorrhages [10]. The Difficult Airway Society and the Association of Anesthetists published a new guideline on how to implement human factors into anesthesia. One of the twelve recommendations is to provide NTS training [11]. This training can be delivered through simulation training, in-theatre training, classroom training, and e-learning [1].

Behavioral rating systems are observational assessment tools that link latent non-observable skills to an observational work task, i.e., a behavioral marker, which is an aspect of effective or ineffective performance [12]. An example of a behavioral marker of good performance in the element of gathering information is that NA obtains patients’ information pre-operatively in a structured way [13]. There are two main types of behavior rating systems, either rating individuals or teams [14]. The first and most widely used behavior rating system for individual NTS in anesthesia is the anesthetists’ non-technical skills (ANTS) [15]. A review by Boet et al. [16] came to the conclusions that the psychometric properties of ANTS are well-tested and found valid and reliable. ANTS have been translated and modified for use among Danish NA, known as nurse anesthetists’ non-technical skills (NANTS), since NTS is also an important skill of the NA work. NANTS-dk consists of 4 categories and 15 elements with examples of good and poor behaviors [17], There is a need for training and evaluating NANTS among Swedish NA, both during their education to become NA and among skilled NA. As there is no Swedish version of NANTS, the aim of this study was to translate and adapt NANTS-dk into Swedish and test its psychometric qualities among Swedish nurse anesthetists who had experience in teaching nurse anesthetist students and junior NAs in clinical settings.

## Material and method

**Fig. 1:**
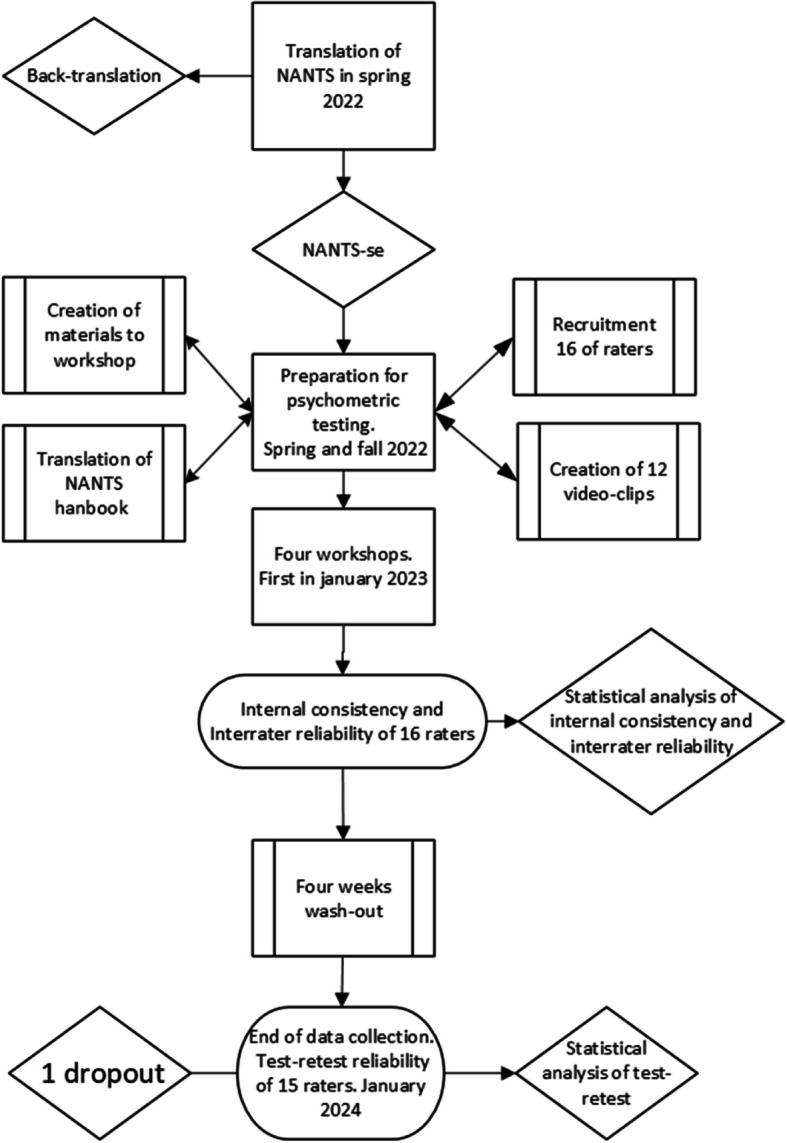
Flowchart of workflow of the study

The design of the study was a prospective psychometric evaluation that included three phases I) preparation; II) translation; and III) internal consistency, interrater reliability, and test–retest reliability of respectively elements in NANTS-se (Table 1), Fig. 1 flowchart of workflow the study.

**Table 1:** Back-translated NANTS-se

Category	Element
Situational awareness	Gather information
	Recognize and understand the situation
	Anticipate and think ahead
Decision making	Identify courses of action
	Assess and balance different courses of action
	Re-evaluate decisions
Work task management	Plan
	Prioritize
	Use resources
	Maintain policies and procedures
Teamwork	Exchange information
	Value the team’s competence
	Coordinate activities
	Show authority, when required
	Demonstrate team-oriented behavior and support team members

I) Preparation

The preparation phase (February 2022 - September 2022) included the translation of the handbook and the creation of video clips. The handbook to NANTS-se is based on the original handbook i.e. ANTS [13] and the English version of the NANTS-dk handbook as reference materials of behavioral markers for nurse anesthetists [18]. The ANTS handbook was chosen as it was more comprehensive in its content compared to the English version of NANTS-dk. However, the NANTS-dk handbook was used as a complement to adapt the behavioral markers in NANTS-se to compare the difference between ANTS and NANTS i.e. the difference in responsibilities between NA and anesthetists. The translation of the handbook was performed by MJ (NA and doctoral student) and reviewed by HC (RN, PhD, researcher in education science) and CE (Anesthetist, PhD and educator at a simulation centre). A review and adaptation of the behavioral markers for each of the elements were done by MJ and CE, to ensure that the elements fit/work in a Swedish context.

Twelve video clips of routine and emergency anesthesia care were created to test NANTS-se psychometrically. The videos were recorded by MJ and CE in a simulated environment using Laerdal SimMan 3G© and Maquet Flow-I© Anesthesia Machine. Three nurse anesthetists were recruited as actors and staff from the sim center filled the roles of other members of the operating room team. The recorded scenarios were edited and cut in Adobe Premier Rush© to create the video clips by MJ. The video clips varied between 1 m 22 sec to 7 m 03 sec in length and were reviewed by UN (NA, PhD, Professor and educator in the nurse anesthetist program) and CE. The number of cases i.e. video clips are based on earlier psychometric testing of ANTS and NANTS [15, 17, 19–22]. In Sweden, it is customary for two anesthesia providers to be present during anesthesia induction, either a NA and an anesthetist, or two NAs. In clinical practice, one anesthesia provider is designated as the primary provider while the other is designated as the secondary provider and assists the primary provider. In eight out of twelve video clips, the primary provider was the focus. Each video clip had a brief description of the case and which provider to observe with a picture of that provider. Only one provider was observed in each video clip. The video clips exhibit a range of behaviors related to non-technical skills, some of good quality and some of poor quality, but most of them display average behavior.

II) Translation

The Consensus-based Standards for the Selection of Health Measurement Instruments (COSMIN) were utilized to guide the translation of NANTS; however, it wasn’t possible to have the original developers as part of the team, as they did not understand Swedish [23]. The first step was translating the English version of NANTS-dk into Swedish, i.e. NANTS-se, by an ISO 17100:2015 certified translator agency. The English version of NANTS-dk was used as none of the researchers understood Danish. Professor Rhona Flin, the developer behind ANTS, gave her permission to translate NANTS-dk into Swedish.

An expert committee consisting of six experts was recruited, including individuals with expertise in anesthesia, simulation situations, non-technical skills, and bilingualism in Swedish and English. This committee evaluated and adapted the instrument for use in a Swedish context and validated its translation into the Swedish language. This was to ensure that the phrases have the same meaning (semantic equivalence) and that the construct has the same meaning (conceptual equivalence). The cultural adaptation of the categories and elements was discussed during individual meetings. Moreover, the expert committee also ensured that phrases of words with a specific meaning not understood by the individual words (idioms) were translated correctly (idiomatic equivalence). To ensure experiential equivalence, an NA (MJ) and anesthetist (CE) discussed the differences in responsibilities between NA and anesthetists in Sweden compared to Denmark, and Scotland where ANTS was developed.

The final NANTS-se was then back-translated to English by a translation agency and thereafter compared with the English version of NANTS-dk and with ANTS. Although there were some minor differences in the wording of the English version of NANTS-se compared to the English versions of NANTS-dk and ANTS, no changes were made.

III) Internal Consistency, Interrater Reliability, and Test-retest Reliability

For the psychometric testing, a convenience sample of 16 NAs (hereafter referred to as raters) was recruited. The raters had experience supervising NA students and junior NAs in clinical settings or with teaching experience.

### Workshops

The raters received an email containing study information and the NANTS-se handbook approximately two weeks before the 3-h workshop. They were asked to read the documents before attending the workshop. Four workshops were conducted by MJ, with two to six raters in attendance each time. The workshop covered the aspects of how to use behavioral rating systems; the categories and elements of NANTS-se; the rating scale of NANTS-se (Table 2). At the workshop each level of the scale was discussed and clarified, including the rating “Not Relevant”. The workshop also covered different rater biases such as halo effect, visceral bias, central tendency, leniency, severity, and contrast effect. Four video clips were used for the raters’ calibration. The video clips were viewed and rated individually, later the raters had a group discussion about their different ratings. At the end of each workshop, the raters received two envelopes: one containing NANTS-se and a demographics survey, and the second containing the NANTS-se retest and evaluation survey.

**Table 2:** Rating scale and description of performance for each rating

Rating	Description
5—Excellent	• Extremely good performance of high professional standard; could serve as a model example for others
4—Good	• Performance of uniformly high standard that enhances the safety of the patient
3—Acceptable	• Satisfactory performance, but can be improved
2—Marginal	• Performance gives rise to concern; improvements required
1—Poor	• Performance exposes the patient to danger or is potentially dangerous for the patient’s safety • Absence of behavior required by the situation the situation
NR – Not Relevant	• Behavior not required in the situation

### Rater instructions

After the workshop, on the same day, an email containing the links to twelve video clips was sent to the raters. To prevent rater fatigue, the raters were advised to watch the video clips in small batches rather than all at once. The raters were told that they could watch the video clips as many times as they needed to make a rating and to record the number of times they watched each video clip. The raters evaluated the skills of individual elements using a five-point scale rating system with performance ranging from 1–5 and “Not Relevant” (Table 2). Additionally, the rating form allowed the raters to leave comments about their choice. After the end of each video clip, the NANTS rating was conducted. Following a four-week washout period, raters were sent an email with links to five of the original clips for retesting. The video clips were named numerically and provided in the same order in the email to all the raters. Table 3 presents the number of days it took to complete each part of the rating and how many times the raters watched each video clip.

**Table 3:** Demographics of the raters (*n* = 16)

	*n* (%)	Median (IQR)
Age (In years)		42 (10)
Sex		
Male	7 (43.7)	
Female	9 (56.3)	
Academic degree		
Bachelor	1 (6.3)	
Masters (1-year)	14 (87.5)	
PhD	1 (6.3)	
Work experience as a NA (in years)	16 (100)	10 (12)
Days to complete part 1		11.5 (15)
Days to complete retest		24 (31)
Times watched each video clip: part 1		1 (0)
Times watched each video clip: retest		1 (0)

*Abbreviations*:* NA* Nurse Anesthetist, *PhD* Doctor of Philosophy

### Rater evaluation

In the rater evaluation, participants were asked four yes-or-no questions and encouraged to provide comments to explain their answers (Table 4). The rater evaluation also included two general free-text answers. One was whether anything was missing in NANTS-se, and the other was if the raters had any additional comments about NANTS-se.

**Table 4:** Rater evaluation

Question	*n*	Yes	No
1. Do you deem that it is easy to do an assessment (rating) with NANTS-se?	16	6	10
2. Do you consider NANTS appropriate for rating nurse anesthetists’ non-technical skills?	16	15	1
3. Do you consider that there is an appropriate number of scale steps for the assessment (rating) of nurse anesthetists?	15	13	2
4. Do you consider that there is an appropriate number of scale steps for the assessment (rating) of nurse anesthetist students?	14	10	4

### Analysis

Descriptive statistics were presented with median and interquartile range (IQR). To analyze internal consistency Cronbach Alpha was calculated for each category to assess the agreement, with a range of 0.70 to 0.95 considered acceptable [24]. Inter-rater reliability was assessed using the intraclass correlation coefficient (ICC) with a two-way random effects model and absolute agreement. Average measurements are reported according to Koo and Li [25], which provide the following interpretation of ICC values: a coefficient less than 0.5 is considered poor, 0.5 to 0.75 indicates moderate reliability, 0.75 to 0.9 is viewed as good, and a value greater than 0.9 signifies excellent reliability. Test–retest reliability was calculated with ICC two-way mixed effect and absolute agreement, and average measurements are reported in accordance with Koo & Li’s guidelines [25]. “Not Relevant” was treated as 0 for statistical analysis, in line with Flynn et al. [20]. During the initial data review, we discovered that only three raters had provided ratings on the category level. This led us to exclude the category rating analysis early on. Missing data on element level was investigated, and according to Schafer [26], an amount of less than 5% is usually seen as small, and a single imputation method is accurate to use. In the present study, missing values were observed in 0% to 4.1% ratings on the element level and significantly impacted the statistical analyses. The missing data was spread across different cases, resulting in the deletion of up to 27.8% of the cases when analyzed with ICC. Therefore, an imputation was performed using the median values for each element. SPSS version 28 was used for the statistical analysis.

The open-ended comments were analyzed following a descriptive approach [27]. The material under each of the six open-ended questions were initially read multiple times to understand the content. Passages were extracted into a common document, codes were identified, and sorted, and three categories were constructed.

## Results

Out of 16 raters, nine were women and seven were men. The median age of the raters was 42, and they had an average of 11 years of experience as NA (Table 3). All raters completed the first test, but one dropped out during the re-test due to misunderstandings and a high workload.

### Rating “Not Relevant”

“Not Relevant” was used in 13.7% of all the observations. During the analysis of the usage of “Not Relevant”, it was found that there was a variation in the use of it where one rater did not use “Not Relevant” at all, while three raters used it in more than 30% of their ratings. “Not Relevant” was shown to be used more frequently when rating NANTS in the short video clips, i.e. clips < 4.5 min compared to clips with a duration > 4.5 min, as well as in video clips that focused on the secondary provider compared to the primary provider. Table 5 displays the rating “Not Relevant” in the video clips by length and provider, supplement materials 1 visual representation of “Not Relevant” sorted on raters.

**Table 5:** Rating “Not Relevant” in the video clips displayed by length and provider

Rated	Length of video clip (minutes)	Category length of video clip^1^	Not Relevant (%)
Primary	7:03	Long	5.6
Primary	5:54	Long	11.7
Primary	5:45	Long	6.7
Primary	5:00	Long	17.8
Primary	4:56	Long	5.0
Primary	4:45	Long	6.1
Secondary	4:05	Short	21.7
Primary	3:13	Short	20.6
Primary	2:15	Short	23.9
Secondary	2:03	Short	15.0
Secondary	1:45	Short	36.7
Secondary	1:22	Short	47.8

^1^The cut between short and long video clips was 4.5 min, i.e. short is less than 4.5 min

### Internal consistency

Acceptable internal consistency was found for NANTS-se, with Cronbach’s alpha ranging between 0.78 and 0.86. The highest reliability was found in Decision Making, while the lowest was found in Work Task Management (Table 6).

**Table 6:** Internal consistency

Category	Cronbach alpha
Situation Awareness	0.81
Decision Making	0.86
Work Task Management	0.78
Teamwork	0.84

### Interrater reliability

Interrater reliability with ICC indicated good reliability, with values ranging between 0.79 and 0.86. The lowest value of ICC was noticed in Situation Awareness, and the highest in Teamwork (Table 7).

**Table 7:** Interrater reliability

Category	ICC	95% Confidence interval	
		Lower	Upper
Situation Awareness	0.79	0.68	0.88
Decision Making	0.83	0.73	0.90
Work Task Management	0.81	0.72	0.88
Teamwork	0.86	0.76	0.89

### Test–retest reliability

Test–retest reliability indicates poor to moderate reliability, with an ICC value ranging from 0.41 to 0.68, with the highest ICC in Decision Making and the lowest in Situation Awareness (Table 8).

**Table 8:** Test–retest reliability

Category	ICC	95% Confidence interval	
		Lower	Upper
Situation Awareness	0.41	0.23	0.54
Decision Making	0.68	0.58	0.76
Work Task Management	0.64	0.55	0.71
Teamwork	0.55	0.45	0.63

### Rater evaluation

The majority of the raters (10 out of 16) responded “No” if they found it easy to rate with NANTS-se. All raters, except for one, agreed that NANTS-se was a suitable behavioral rating system for rating NTS among NA. The one who disagreed commented that the system is only for rating the primary provider (Table 4). The three categories from the open-ended questions were: *A new tool, The video clips, and cognitive skills*.

*A new tool.* As NANTS-se was a new tool for the raters, they found it somewhat complicated to use even though some said there were clear instructions on how to use NANTS-se. In the first video clips the raters found that NTS was difficult to rate, but it became easier as they continued to make ratings. Several raters expressed that rater training is needed and that raters need to use NANTS-se on a regular basis. The raters expressed that since they were unfamiliar with the definitions of the elements, they suggested it might be easier if several raters were to observe and discuss to conclude on a rating as they did in the workshop. At the same time, other raters expressed that the division into elements made it easier to identify and improve weak spots in NA. The usability of NANTS-se was expressed with the quote:

“It’s easier than normal but still difficult!” (Rater 13)

*The video clips*. The raters found that ratings of NTS in short video clips were more difficult since they didn’t have enough information about the situation. The raters also found rating video clips difficult when there were both very good and very poor behaviors occurred in the same element. The raters also found that it was easier to rate the primary NA. Ratings of short video clips was expressed with the quote:

*“In longer scenarios, it is easier since you grasp the whole picture. In short scenarios, you lack too much information for it to be good.”* (Rater 6)

*Cognitive skills.* Raters expressed that it could be challenging to assess cognitive skills in NTS because they can’t be observed, and a rater expressed the difficulty with the quote:

*“It is always difficult to assess things that are not concrete, for example, it is easier to assess or help someone who has incorrect technique during intubation* (technical skills for placing a tube to help the patient breathe during anesthesia: authors explanation).” (Rater 8)

## Discussion

The initial testing of NANTS-se has shown good internal consistency, good inter-rater reliability, and poor to moderate test–retest reliability. This indicates that NANTS-se is a reliable tool for measuring an NA´s non-technical skills. These findings are in line with the initial testing of previous studies on behavioral rating systems for NA and anesthetists [15, 20].

The high amount of “Not Relevant” ratings, especially in the short clips, < 4.5 min, and when rating the secondary provider was unexpected. The challenge to rate assistants (secondary providers) can be explained by the fact that the original ANTS tool was developed to assess only the primary anesthetists. Regarding the length of video clips, capturing all elements of NTS in a short video clip can be challenging. However, short video clips have the advantage of not requiring raters to address performance changes over time. In a study by Reim et al. [28], they use five-minute-long video clips and argue whether it is possible to assess all elements of ANTS during a short video clip. In the present study, “Not Relevant” was never agreed upon in any of the elements in the video clips by the raters. This may be explained by “Not Relevant” as a “safe card” when the raters feel that ratings are difficult or that the raters did not notice an observable behavior. Williams et al. [29] compare the ratings of figure skating judges in the Olympics. These judges have at least 15 years of experience, are highly trained, use clearly defined criteria for assessment, and get continuous feedback on their ratings compared to others. Even if these judges have a high degree of agreement, it still ranged from 0.93–0.97. This example indicates how much training and experience it takes to calibrate an assessment and still not reach 100% agreement. Even if the raters in the present study have experience teaching NA students and junior NA in clinical practice, NANTS-se is a new assessment tool and the rater’s experience in NANTS-se is by no means close to the experience or training of judges in figure skating. However, if NANTS-se is to be implemented in education and clinical practice, the level of agreement should increase as the raters gain more experience.

The raters agreed that NANTS-se is an appropriate behavioral rating system to rate the primary NA, even though the raters find NANTS-se to be somewhat complicated to use. The raters had suggestions on how to make rating easier, such as continuous education, frequent use of NANTS-se in clinical practice and education, or if several raters observed the same case and discussed to come to a conclusion on a rating. The ratings of the NTS might been easier if ratings were conducted directly after the workshop since the information from the calibration workshop was fresh in memory. However, this was not possible because the nurse anesthetist could not take time off work for several hours due to a heavy workload in the anesthesia department after the pandemic. Additionally, the risk of rater fatigue was considered. The present study’s poor to moderate test–retest reliability shows that a four-week washout period seems to be sufficient. The difference in ICC scores, with Situation Awareness having the lowest and Teamwork having the highest, can be attributed to the fact that Situation Awareness is a cognitive skill while Teamwork is a social skill. This makes Situation Awareness more difficult to observe than Teamwork, which mainly relies on communication between team members. That raters feel uncertainties when rating NTS is not an exclusive challenge when using a behavioral rating system. Tweed et al. [30] investigated examinators’ confidence in grading students and found that examinators were less confident when it came to failing than to pass a student. The raters in our study considered NANTS-se to be an aid to assess NA students who are on the borderline of pass or fail, since NANTS-se puts words on the skills needed in anesthesia nursing. The challenges of supervising NA students in clinical education are described by Hedlund et al. [31]. They found that the clinical supervisors emphasized the importance of identifying students at risk of not achieving their educational goals. In the present study, the raters found that NANTS-se is a valuable tool for identifying and improving weak aspects of NTS.

The ANTS behavioral rating system does not have a uniform scale level. For instance, while ANTS has four levels as described by Fletcher et al. [15], NANTS-dk [32], and NANTS-no [20] have five levels. We decided to continue using the five level scale used in Denmark and Norway. This is to ensure consistency for future comparisons as the education required to become an NA and the clinical competence are in these two countries as compared to Sweden.

It was challenging to record clips with poor behavior because when the actors were asked to underperform, the whole anesthesia simulation became poor. Lyk-Jensen et al. [32] note high interrater reliability before training NTS and argue that it is because of too easy video clips displaying only poor or good performance. In the present study, the “best” and most realistic display of poor performance was due to the actor’s mistake during the recordings, thereby displaying a more realistic behavior.

The literature on how to design a psychometric evaluation of behavioral rating systems is scant. Therefore, we used other research in the field of behavioral rating systems of non-technical skills [15, 17, 20] to guide the design, number of raters, and methodology of this study. We notice challenges with using this approach and the one option would be to use a smaller number of raters with an increased number of video clips; on the other hand, more raters can capture a larger variety of opinions and make the results more valid.

In the current study, missing data is less than 5%, but it still significantly impacted the analysis of the data with a loss of 27.8% of the observations since the missing data are spread out through the observations. The high amount of data loss occurs because if any data is missing in a row, the entire row is excluded. Some researchers argue that missing data less than 5% is inconsequential [33], but as seen in the present study, missing data needs to be inspected and not just generalized. Therefore, we decided to impute the median value since it still is a low amount of missing data, and according to Schafer [26], a single imputation method can be accurate. We ran analyses with mean values to see how they affected the results, but there was only a slight difference in ICC on Teamwork; all other values were the same as those of Median. However, how to handle the rating “Not relevant” has been a challenge. An imputation with a minimum value of 1, a maximum value of 5, and a mean was carried out, resulting in slightly higher ICC values for inter-rater reliability. Interestingly, when testing with imputation using expectation maximization, it resulted in the highest ICC values of all imputation methods. However, the decision was made to impute 0, as this value had been used by previous researchers [20].

### Limitations

This research utilized a convenience sample consisting of raters with prior assessment experience. Previous studies [15, 20, 32] employed reference ratings; however, due to the absence of a gold standard for NTS in Sweden, we could not include reference ratings in this study. Further research to establish a gold standard for NTS in clinical practice would be desirable. Additionally, further validation of NANTS-se across clinical settings, educational contexts, and different geographical areas would be beneficial.

## Conclusion

By using NANTS-se, the non-technical skills required in anesthesia nursing are articulated. The initial testing of NANTS-se shows good Internal consistency, inter-rater reliability, and moderate test–retest reliability for assessing primary anesthesia providers. This gives a foundation for future research on NANTS-se, both as a tool for assessing NA students’ NTS and the clinical performance of NA. However, there are some challenges to consider in future research; the handling of “Not Relevant” needs to be addressed, as well as the best length of video clips for the observation.

## Supplementary Information

Supplementary Material 1.

Supplementary Material 2.

## Data Availability

The datasets generated and/or analyzed during the current study are not publicly available due to ethical permissions for the study but are available from the corresponding author upon reasonable request.

## Abbreviations

ANTS: Anesthetist non-Technical Skills
COSMIN: The Consensus-based standards for selection of health measurement instruments
ICC: Intraclass Correlation Coefficient
IQR: Interquartile Range
NA: Nurse Anesthetist
NANTS: Nurse Anesthetist Non-Technical Skills (ending indicating country)
OR: Operation Room
