# Supplementary material for: Psychometric evaluation of a structured assessment tool for nurse anesthetists’ non-technical skills
Source: BMC Med Educ. 2025 May 17;25:718. doi: 10.1186/s12909-025-07297-2 (PMC12085836; doi:10.1186/s12909-025-07297-2)
Supplement: Supplementary file 1 — Supplementary Material 1. [file 12909_2025_7297_MOESM1_ESM.docx]

| Category & category ratings (1-5*) | Element | Element ratings (1–5*) | Notes |
| --- | --- | --- | --- |
| Situational  awareness | Gather information |  |  |
|  | Recognize and understand the situation |  |  |
|  | Anticipate and think ahead |  |  |
| Decision making | Identify courses of action |  |  |
|  | Assess and balance different courses of action |  |  |
|  | Re-evaluate decisions |  |  |
| Work task management | Plan |  |  |
|  | Prioritize |  |  |
|  | Use resources |  |  |
|  | Maintain policies and procedures |  |  |
| Teamwork | Exchange information |  |  |
|  | Value the team’s competence |  |  |
|  | Coordinate activities |  |  |
|  | Show authority, when required |  |  |
|  | Demonstrate team-oriented behavior and support team members |  |  |

*1 – Poor, 2 – Marginal, 3-Acceptable, 4 – Good, 5 – Excellent, NR – Not Relevant
